# Evaluating the performance of DeepSeek-V3, Doubao, ERNIE 4.5 Turbo and iFLYTEK Spark in the Chinese national nursing licensing examination: a cross-sectional comparative study

**DOI:** 10.3389/frai.2026.1870847

**Published:** 2026-08-17

**Authors:** Min Yang, Li Zhu, Zeju Zhang, Qigui Xu

**Affiliations:** 1 School of Nursing, Chongqing Medical and Pharmaceutical College, Chongqing, China; 2 School of Pharmacy, Chongqing Medical University, Chongqing, China

**Keywords:** large language models, Chinese nursing licensing examination, artificial intelligence, AI, cross-sectional design

## Abstract

**Background:**

While large language models (LLMs) have been widely adopted in nursing education and several studies have evaluated their performance on the Chinese National Nursing Licensing Examination (NNLE), systematic comparisons focusing on the latest generation of Chinese LLMs such as DeepSeek-V3, Doubao, ERNIE 4.5 Turbo, and iFLYTEK Spark remain limited. Furthermore, no study has specifically examined the multimodal capabilities of these models using image-based nursing questions.

**Objective:**

This study aimed to compare the performance of DeepSeek-V3, Doubao, ERNIE 4.5 Turbo, and iFLYTEK Spark LLMs on the NNLE and evaluate their potential for nursing education.

**Methods:**

This cross-sectional study assessed DeepSeek-V3, Doubao, ERNIE 4.5 Turbo, and iFLYTEK Spark using questions from the 2025 NNLE, including both authentic text-based items (*n* = 240) and image-based simulation items (*n* = 80). We evaluated model performance across several dimensions, including overall accuracy, accuracy by question type (A1, A2, A3, A4), accuracy on case analysis versus non-case analysis questions, accuracy on text-based versus image-based questions and accuracy on disciplinary background (nursing vs. non-nursing). All reported accuracies are presented with 95% confidence intervals. Additional metrics included generation speed, session capacity, accessibility, and content modality support (text, image).

**Results:**

On text-based NNLE items, all four models achieved accuracy rates above 90% (DeepSeek-V3: 92.9, 95% CI [89.0, 95.6%]; Doubao: 94.6, 95% CI [91.0, 96.9%]; ERNIE 4.5 Turbo: 93.3, 95% CI [89.5, 95.9%]; iFLYTEK Spark: 92.1, 95% CI [88.0, 95%]), meeting the NNLE passing requirement. No significant differences in overall accuracy were observed among models (all pairwise comparisons *p* > 0.05). On image-based simulation items, performance declined substantially across all multimodal-capable models (Doubao: 48, 95% CI [37.2, 58.9%]; ERNIE 4.5 Turbo: 48, 95% CI [37.2, 58.9%]; iFLYTEK Spark: 61, 95% CI [50, 71.4%]), with DeepSeek-V3 unable to process image inputs due to its text-only architecture. For the image-based simulation test items, the performance difference among the three models was not statistically significant (χ^2^ = 4.040, *p* = 0.133). The difference between text-based and image-based performance was statistically significant for all models (*p* < 0.01). Subgroup analyses based on question type, case status, and disciplinary content revealed variable performance patterns, though these findings should be interpreted with caution given limited sample sizes in certain categories.

**Conclusion:**

This study demonstrates that DeepSeek-V3, Doubao, ERNIE 4.5 Turbo, and iFLYTEK Spark perform well on Chinese nursing examinations questions in text format, indicating their potential as auxiliary resources for nursing education and exam preparation. However, their performance on image-based items is substantially lower, and the findings do not support claims of clinical applicability. Further research is needed to assess their utility in real-world clinical reasoning or patient care.

## 1 Introduction

In recent years, the rapid advancement of LLMs has presented unprecedented opportunities and challenges for the healthcare sector. China has recognized the profound impact of artificial intelligence (AI) on education and actively promotes its integration into teaching practices to foster further innovation and transformation in the field (Lu et al., 2023). AI technology is currently being extensively utilized in key areas of nursing in China, including nursing education, clinical decision-making, nursing management, and risk assessment (Li, 2025; Zhang et al., 2026; Wang et al., 2024; Fang et al., 2025). Among these applications, notably DeepSeek-V3, leverage their powerful natural language processing capabilities and have shown significant promise in supporting nursing education, analyzing nursing texts, supporting clinical decision-making, and assisting research efforts (Huang et al., 2025; Feng et al., 2025; Zeng et al., 2026; Zhou et al., 2025).

Nursing, a discipline that combines theoretical knowledge with practical complexity, demands high-level comprehensive competency from students (Tam et al., 2023). NNLEs are a critical entry test for nursing licensure, and successfully passing them indicates a foundational understanding of nursing knowledge. A model’s ability to pass these exams not only suggests its potential to aid nursing students in their exam preparation but also reflects its proficiency in nursing knowledge and reasoning-skills essential for clinical decision-making. Against this backdrop, exploring the application potential of LLMs in nursing education, particularly their performance and limitations in standardized examinations, has become a subject of shared interest for both academia and the educational sector.

Previous studies have evaluated the performance of various LLMs on Chinese nursing examinations. Zong et al. (2024a, 2024b) assessed ChatGPT on the NNLE from 2017 to 2021, and found that it failed to pass the examination. Zhu et al. (2025) evaluated seven LLMs, including GPT-3.5, GPT-4.0, GPT-4o, Copilot, ERNIE Bot-3.5, SPARK, and Qwen-2.5, on CNNLE questions from 2019 to 2023, finding that Qwen-2.5 achieved the highest accuracy of 88.9%. Xu L. H. et al. (2025) and Xu W. B. et al. (2025) examined four LLMs (Sider Fusion, GPT-4o, Gemini 2.0 Pro, and DeepSeek V3) on the 2024 CNNLE, reporting that DeepSeek V3 and Gemini 2.0 Pro achieved overall accuracies exceeding 83%. Li et al. (2025) assessed ChatGPT-3.5, ChatGPT-4, and iFLYTEK Spark on the China National Nursing Professional Qualification Exam, finding that ChatGPT-4 and iFLYTEK Spark performed well. Additionally, Zhan et al. (2026) conducted a three-year longitudinal analysis of 15 LLMs on the NNLE from 2022 to 2025.

Despite these contributions, several gaps remain. First, systematic comparisons of the latest generation of Chinese LLMs, particularly DeepSeek-V3, which has demonstrated strong performance on the National Medical Licensing Examination, Doubao, ERNIE 4.5 Turbo, and iFLYTEK Spark on the NNLE are scarce. Second, previous studies have primarily focused on text-only questions, with limited attention to multimodal performance on image-based nursing questions. Third, the user experience dimensions (generation speed, session capacity, accessibility, and content modality support) of these models as potential instructional tools have not been systematically compared. This study utilizes a dataset of 2025 NNLE questions, supplemented by an image-based simulation set, to systematically analyze and compare the performance of four leading Chinese LLMs across multiple dimensions, including accuracy, question-type adaptability, multimodal capability, and usability. This work provides a comparative evaluation of these recently released Chinese proprietary models on the 2025 examination, with a novel focus on image-based question performance and detailed subgroup analyses across multiple question categories. This study aims to provide an evidence base for the appropriate application of these AI models in nursing education in China.

## 2 Methods

### 2.1 General information

This study employed a cross-sectional design. We selected several mainstream generative AI models currently prominent in China for evaluation. The included models were DeepSeek-V3, Doubao, ERNIE 4.5 Turbo, and iFLYTEK Spark. These models were developed by leading Chinese AI companies: DeepSeek-V3 by DeepSeek, ERNIE 4.5 Turbo by Baidu, Doubao by ByteDance, and iFLYTEK Spark by iFLYTEK.

We selected these four models to represent distinct institutional and technical approaches among Chinese LLMs: DeepSeek-V3 (text-only architecture), ERNIE 4.5 Turbo (multimodal), Doubao (general-purpose), and iFLYTEK Spark (Chinese-language optimized). At the time of study initiation, they were among the most widely used and freely accessible Chinese LLMs in educational settings. Earlier versions of some models such as ERNIE Bot-3.5 at 78.1% and SPARK at 65.0% on the CNNLE, have shown disparate performance, prompting assessment of their updated releases.

### 2.2 NNLE description

The NNLE is a standardized, nationwide test required for nursing licensure in China. The examination consists of two sections: Professional Practice and Practice Competence. The Professional Practice section assesses foundational nursing knowledge (e.g., disease mechanisms, core nursing theories, and psychosocial applications). The Practical Competence section evaluates clinical application, including health assessment, intervention planning, clinical techniques and procedures, and patient education. It is administered in four half-day sessions, with candidates randomly assigned to one session to complete both sections. Each section contains 120 multiple-choice questions, resulting in a total of 240 items. The exam is computer-based and uses an interactive format, presenting objective questions in various forms, including text, images, and videos. All questions are in Chinese and follow a multiple-choice structure with five options, only one of which is correct. The total examination duration is 100 min. To pass, candidates must score at least 300 points in each section.

The questions in the exam are classified into 4 types. A1-type questions assess basic nursing knowledge, A2-type, A3-type, and A4-type questions are case analysis questions. The A2-type questions are presented with a single case, followed by 1 question related to that case. A3 and A4-type questions also follow a case-based format: each case is accompanied by 2 to 4 associated questions. These questions assess single knowledge points, analysis and judgement skills, nursing problem- solving and comprehensive clinical ability.

Statistical data from 2011 to 2021 show that the pass rate for first-time test-takers in the National Nurse Practitioner Qualification Examination remained stable, ranging between 70 and 74%. Among these candidates, those with a bachelor’s degree consistently achieved a pass rate of approximately 97%, whereas for graduates from vocational colleges, the pass rate held steady between 81 and 84% from 2015 onward (You and Fu, 2022). Over the past five years, the overall pass rate for all candidates has ranged from 60 to 65% (Health Human Resources Development Center, National Health Commission, P.R. China, 2025). In contrast, some provinces reported pass rates falling below 20% during the same period (Shigatse Municipal Health Commission, 2024), highlighting the considerable difficulty of the examination. Research ethics approval was not necessary as no patient data or identifiable information was used.

### 2.3 Test items

This study used test items from the 2025 NNLE, including past papers and simulated questions published by Zhengzhou University Press and People’s Medical Publishing House. For the image-based component, we used simulated questions released in publicly available format by the NNLE Center of People’s Medical Publishing House, the officially designated publisher for examination preparation materials. These simulation items were developed by senior nursing educators with extensive experience in NNLE test preparation, and were designed to match the content, difficulty level, and format of authentic NNLE image-based questions. Selection followed three criteria: (1) clinical relevance, (2) format fidelity to the official NNLE structures, and (3) difficulty calibration by experienced nursing educators. All images were standardized to 1920 × 1,080 pixels (PNG format) and presented in the same order under identical network conditions. Video-based items were excluded due to the current limitations of large language models in interpreting dynamic visual scenarios. These materials are standardized and publicly available, making them suitable for assessing models’ foundational nursing knowledge. All materials are used in compliance with copyright exemptions for academic research. We acknowledge that the simulated nature of the image-based component limits the generalisability of our multimodal findings to authentic NNLE items.

### 2.4 Testing environment and model specifications

All evaluations were conducted on a desktop computer with an Intel Core i7-11700K processor, 32 GB RAM, and an NVIDIA GeForce RTX 3080 GPU, running Windows 11. The web interface was accessed via Google Chrome (version 122.0.6261.129) over a stable broadband connection (download speed ≥100 Mbps, latency ≤20 ms). The specific model versions tested were: DeepSeek-V3 (version v3-202501, accessed at: deepseek.com), Doubao (version 1.2.4, accessed at: doubao.com), ERNIE 4.5 Turbo (version 4.5-turbo-202501, accessed at: yiyan.baidu.com), and iFLYTEK Spark (version spark-3.5-202501, accessed at: xinghuo.xfyun.cn). All models were used through their official web-based interface (rather than API) with default settings; each model was tested with reasoning mode enabled and retrieval-augmented generation (RAG) disabled; the temperature parameter was set to the minimum randomness level (equivalent to 0) wherever the interface allowed. Additionally, we have included the exact prompt template used: “Acting as a nursing education expert, provide the correct answer to each examination items and explain the rationale in language accessible to non-clinical readers”.

### 2.5 Model testing process

All models were evaluated via their public web interfaces in default configurations, without manual parameter adjustment. A standardized prompting approach was adopted, with no in-context learning. A standardized Chinese prompt was copied into the conversation window of each model to establish the test conditions. For A1- and A2-type questions, one question was entered at a time, and models responded individually to each. For A3- and A4-type questions, all questions related to a single case were entered simultaneously, and models provided answers to all questions within that case. Each question set was processed in a new, blank chat session. For models with image recognition capability, images were uploaded directly through the web interface; for DeepSeek-V3, which does not support image input, image-based questions were not administered. A dedicated spreadsheet was used to record each model’s answers and responses. Evaluation was conducted between January 9 and January 23, 2026.

### 2.6 Outcome measure

The test questions and reference answers were sourced from materials published by Zhengzhou University Press and the People’s Medical Publishing House in 2025. The responses generated by the four LLMs were compared against the reference answers to assess their accuracy. All outputs from the models were recorded faithfully by the researchers, who then evaluated the accuracy of each model’s responses and noted instances where no answer was produced. Questions in which a model provided no response were classified as incorrect responses.

### 2.7 Statistical analysis

The performance of the LLMs was assessed based on response count and accuracy rate. Response count was defined as the number of questions each model answered, while accuracy rate was calculated as the proportion of correct responses relative to the total number of questions attempted. These metrics were used to quantify the number of correct answers provided by each LLM for every subject area. Questions that received no response were recorded as omitted and excluded from the corresponding accuracy calculation. A grouped bar chart compared the percentage of correct answers across models and question categories.

For paired-sample comparisons, the McNemar chi-square test was used for binary comparisons, and the Cochran Q test was applied for multiple-group comparisons. Given the exploratory nature of these subgroup analyses, which were intended for hypothesis generation rather than confirmatory inference, we did not apply formal multiplicity adjustments such as Holm or Benjamini-Hochberg correction. For independent-group comparisons, we used the Pearson chi-square test, with continuity correction or Fisher’s exact test applied when expected cell frequencies were less than 5. All statistical analyses were performed using SPSS (version 27.0; IBM Corp), and a two-tailed *p* value of <0.05 was considered statistically significant. Wilson score intervals were calculated to provide 95% confidence intervals for all reported accuracy rates.

## 3 Results

### 3.1 Comparison of ease of use

This study evaluated the user experience of four LLMs across four dimensions: response time, session capacity, accessibility, and content modality support (Table 1). Regarding response time, we measured response latency for a standardized set of 50 text-based questions (mean length:45 characters). Mean response times were as follows: DeepSeek-V3, 12.4 s (SD = 3.1); Doubao, 8.7 s (SD = 2.4); ERNIE 4.5 Turbo, 10.2 s (SD = 2.8); and iFLYTEK Spark, 9.5 s (SD = 2.6). All models consistently completed responses within 20 s per question. In terms of session capacity, DeepSeek-V3 supported the highest number of queries per session (120), followed by iFLYTEK Spark (100), ERNIE 4.5 Turbo (90), and Doubao (64). For accessibility, all models were freely available without access restrictions. In the dimension of content modality, ERNIE 4.5 Turbo supported the widest variety of content types (text, image, audio, video), whereas DeepSeek-V3 was limited to text-only input and output.

**Table 1:** Comparison of user experience metrics across four LLMs.

Metric	DeepSeek-V3	Doubao	ERNIE 4.5 Turbo	iFLYTEK Spark
Mean response time (seconds)	12.4 ± 3.1	8.7 ± 2.4	10.2 ± 2.8	9.5 ± 2.6
Max questions per session	120	64	90	100
Free access	Yes	Yes	Yes	Yes
Access restrictions	None	None	None	None
Query limit	Yes	Yes	Yes	Yes
Supported modalities	Text only	Text, Image	Text, image, audio, video	Text, image, audio

### 3.2 Classification of test items

The test items were analyzed according to four dimensions: stem information, content assessed, content format, and question type. In terms of item information, the questions were nearly evenly split between case-based and non-case-based formats. Regarding content assessed, the vast majority of items were nursing-related, with only a small number falling into the non-nursing category (exclusively in the professional practice paper). With respect to content format, text-only questions predominated across the authentic exam papers, while all items in the image-based simulation set naturally included visual elements. Finally, based on question type classification, A1-type questions were the most prevalent across all three sets, followed by A2 and then A3/A4. A detailed breakdown of the classification is presented in Table 2.

**Table 2:** Classification of question types in the 2025 NNLE (*N* = 320).

Category	Question type	Test items in the nursing licensure examination		Image-based simulation (*n* = 80)
		Professional practice (*n* = 120)	Practical competence (*n* = 120)	
Item information	Case-based	53	75	32
	Non-case-based	67	45	48
Content assessed	Nursing-related	106	120	80
	Non-nursing	14	0	0
Content format	Text-only	116	118	0
	Text + Image	4	2	80
Item type	A1	67	45	44
	A2	27	50	21
	A3/A4	26	25	15

Non-nursing items include questions on general sciences, ethics, or healthcare management.

### 3.3 Accuracy of test items in the NNLE

#### 3.3.1 Overall accuracy

**Figure 1:**
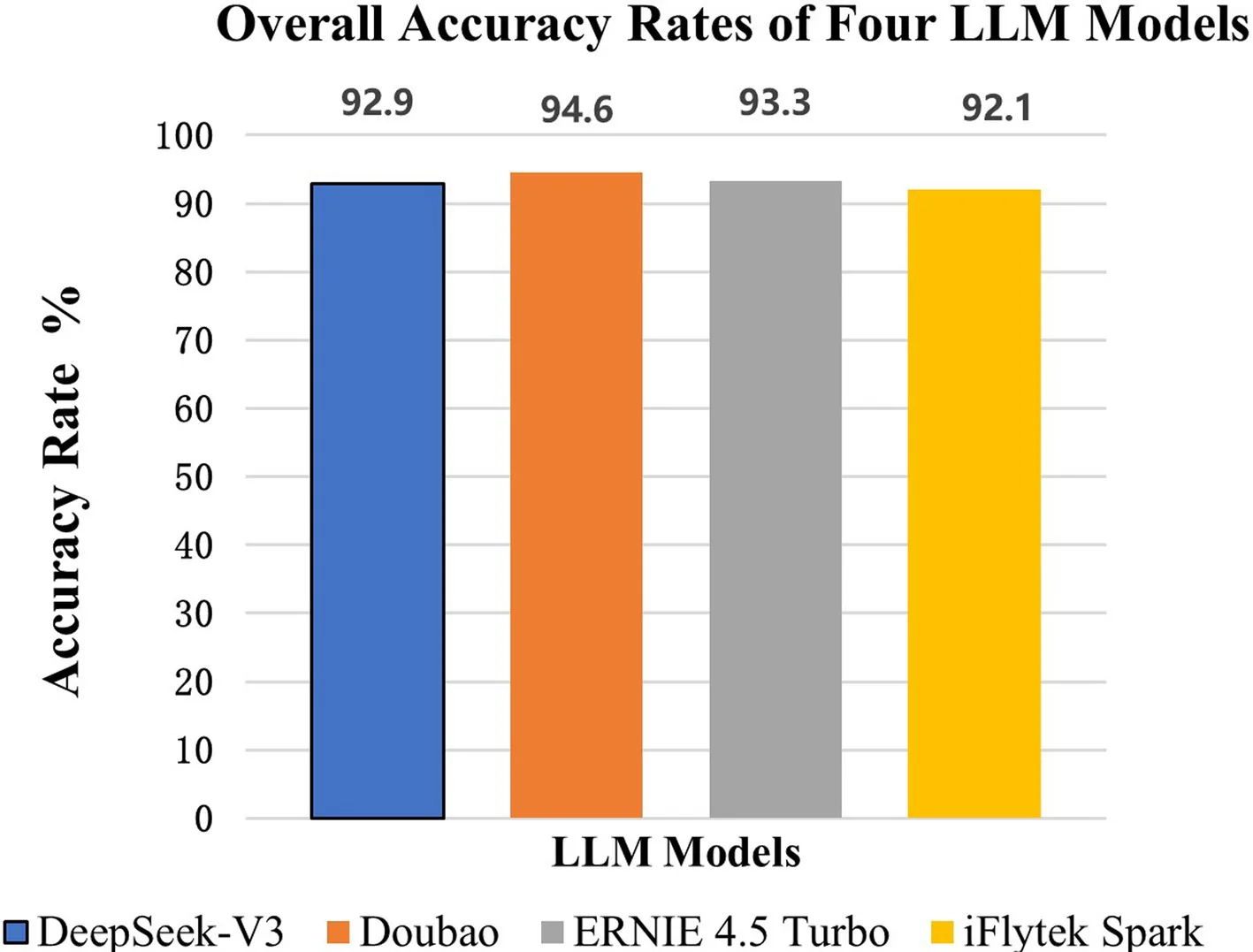
Overall accuracy rates of DeepSeek-V3, Doubao, ERNIE 4.5 Turbo and iFLYTEK Spark.

For the 240 text-only NNLE items (excluding the 6 image-text items), the models answered 223(DeepSeek-V3), 227(Doubao), 224(ERNIE 4.5 Turbo), and 221(iFLYTEK Spark) questions correctly, respectively, achieving accuracy rates of 92.9% (223/240; 95% CI: 89.0–95.6%), 94.6% (227/240; 95% CI: 91.0–96.9%), 93.3% (224/240; 95% CI: 89.5–95.9%), and 92.1% (221/240; 95% CI: 88.0–95.0%) (Figure 1). All models attained accuracy rates above 90%, consequently meeting the passing requirement of the NNLE. No significant differences in overall accuracy were observed across the four models (Cochran’s Q = 1.214, *p* = 0.750). Pairwise comparisons yielded the following risk differences: DeepSeek-V3 vs. Doubao: −1.7 percentage points (95% CI: −6.5 to 3.2); DeepSeek-V3 vs. ERNIE 4.5 Turbo: -0.4 (95% CI: −5.2 to 4.4); DeepSeek-V3 vs. iFLYTEK Spark: 0.8 (95% CI: −4.0 to 5.6); Doubao vs. ERNIE 4.5 Turbo: 1.3 (95% CI: −3.5 to 6.0); Doubao vs. iFLYTEK Spark: 2.5 (95% CI: −2.1 to 7.2); ERNIE 4.5 Turbo vs. iFLYTEK Spark: 1.2 (95% CI: −3.4 to 5.9). The narrow confidence intervals suggest that the absence of statistically significant differences reflects genuine similarity in performance rather than insufficient statistical power. For the image-based simulation items, DeepSeek-V3 could not process any image input; therefore, its performance on these items is marked as not applicable (N/A) rather than as zero correct.

#### 3.3.2 Accuracy across different question types

**Figure 2:**
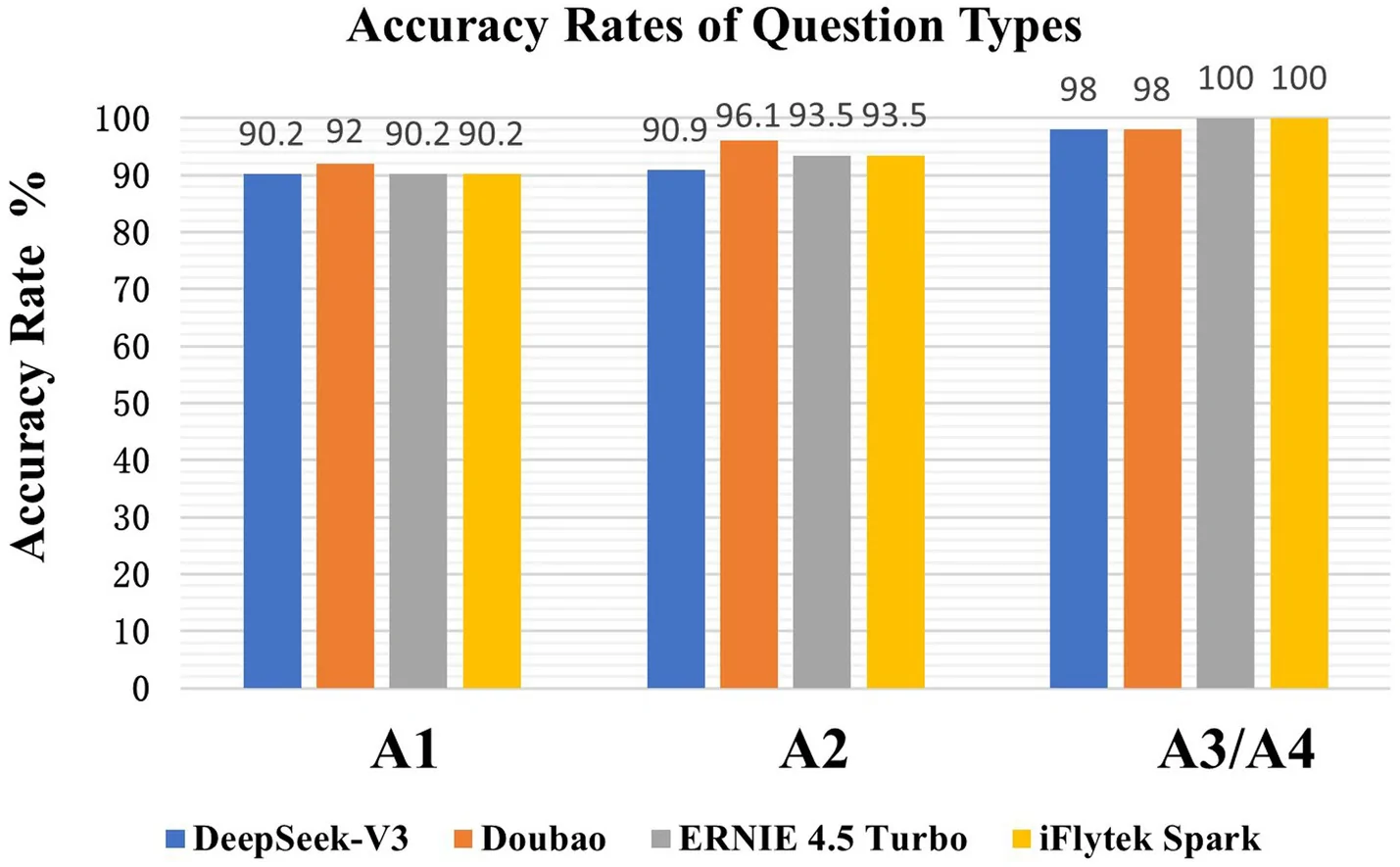
Accuracy rates of DeepSeek-V3, Doubao, ERNIE 4.5 Turbo and iFLYTEK Spark across various question types.

As shown in Figure 2, all four models demonstrated consistently high accuracy across the A1, A2, and A3/A4 question types, each exceeding 90%, with no statistically significant differences observed among them (*p* > 0.05 for all pairwise comparisons). Specifically, for the A1 items, DeepSeek-V3, ERNIE 4.5 Turbo, and iFLYTEK Spark each achieved an accuracy of 90.2% (101/112; 95% CI: 83.3–94.4%), while Doubao recorded 92.0% (103/112; 95% CI: 85.5–95.8%). On the A2 items, DeepSeek-V3 attained 90.9% (70/77; 95% CI: 82.4–95.6%), Doubao 96.1% (74/77; 95% CI: 89.0–98.7%), and both ERNIE 4.5 Turbo and iFLYTEK Spark 93.5% (72/77; 95% CI: 85.7–97.2%). For the A3/A4 items, ERNIE 4.5 Turbo and iFLYTEK Spark achieved perfect scores (100%, 51/51; 95% CI: 93.0–100%), followed by DeepSeek-V3 and Doubao (98.0%, 50/51; 95% CI: 89.7–99.9%). Although the A3/A4 items elicited the highest accuracy overall, within-model comparisons across the three question types also revealed no significant differences (*p* > 0.05).

#### 3.3.3 Accuracy across case analysis and non-case analysis questions

**Figure 3:**
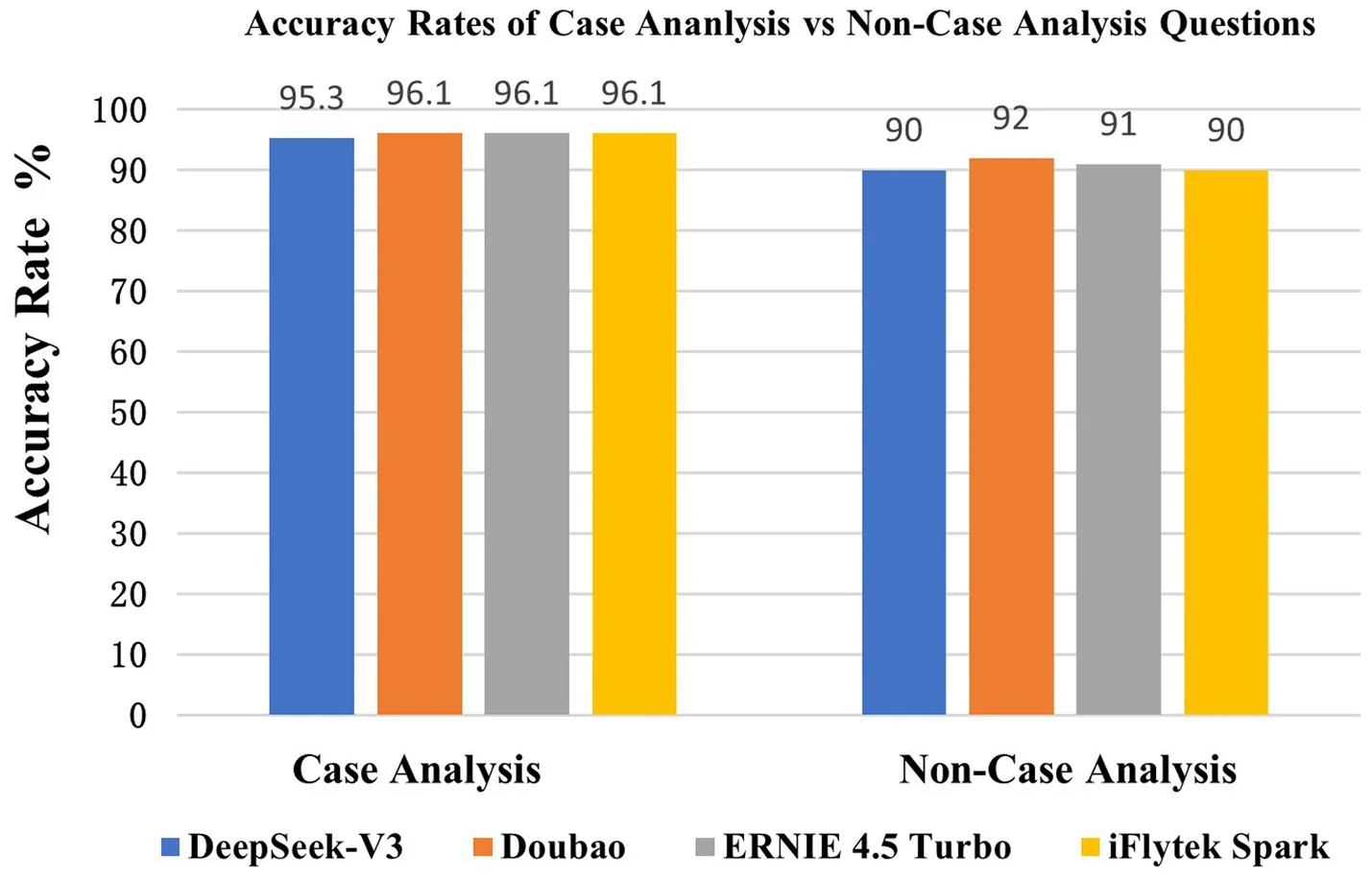
Accuracy rates of DeepSeek-V3, Doubao, ERNIE 4.5 Turbo and iFLYTEK Spark on case analysis and non case analysis questions.

Of the 240 total questions, 128 (53%) were case analysis questions and 112 (47%) were non-case analysis questions. As shown in Figure 3, the accuracy rates for both question types were similar across all four models (DeepSeek-V3, Doubao, ERNIE 4.5 Turbo, and iFLYTEK Spark).

For case analysis questions, DeepSeek-V3 answered 122 of 128 correctly (95.3, 95% CI: 90.0–98.2%). While Doubao, ERNIE 4.5 Turbo, and iFLYTEK Spark each answered 123 correctly (96.1, 95% CI: 91.1–98.5%). Pairwise comparisons showed no statistically significant differences among the four models (*p* = 0.96). Similarly, for non-case analysis questions, no significant differences were found in any pairwise comparison (all *p* > 0.05).

#### 3.3.4 Accuracy across nursing-related and non-nursing questions

**Figure 4:**
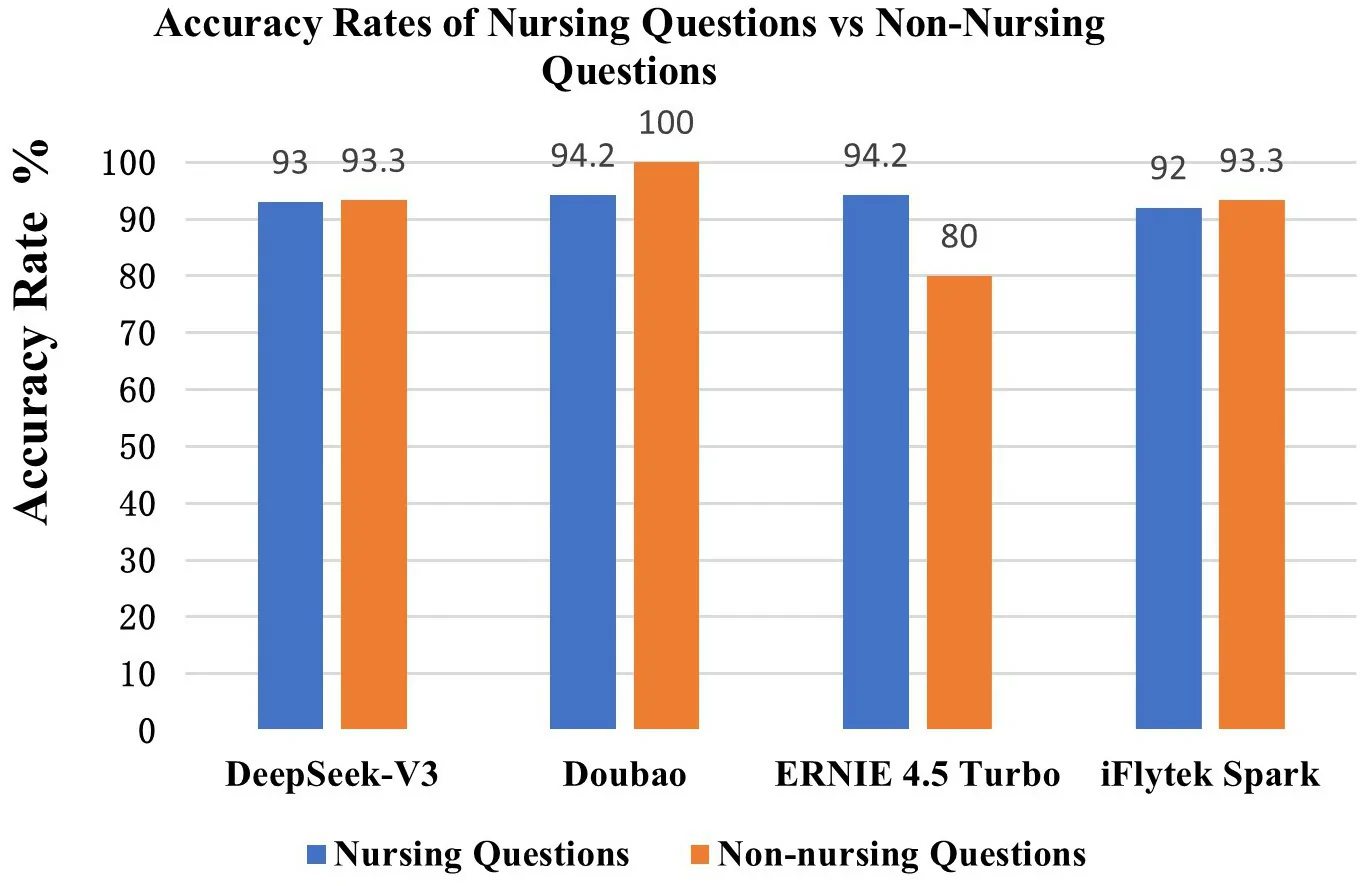
Accuracy rates of DeepSeek-V3, Doubao, ERNIE 4.5 Turbo, and iFLYTEK Spark on nursing-related and non-nursing questions.

Of the 240 questions, 225 (94%) were nursing-related and 15 (6%) were non-nursing questions. As shown in Figure 4, all models performed well on both types, with accuracy rates exceeding 90% on nursing questions and varying more on non-nursing ones. However, due to the small sample size of non-nursing questions (*n* = 15), these subgroup findings should be interpreted with caution and considered hypothesis-generating rather than confirmatory.

For nursing questions, ERNIE 4.5 Turbo and Doubao each answered 212 out of 225 (94.2, 95% CI: 90.4–96.7%), DeepSeek-V3 answered 209 (93, 95% CI: 88.9–95.6%), and iFLYTEK Spark answered 207 (92.0, 95% CI: 87.8–94.9%). For non-nursing items (*n* = 15), Doubao achieved a perfect score (15/15, 100, 95% CI: 79.6–100%), reflecting its strength in general knowledge. DeepSeek-V3 and iFLYTEK Spark each answered 14 correctly (93.3, 95% CI: 70.2–98.8%), showing comparable performance. In contrast, ERNIE 4.5 Turbo answered 12 correctly (80.0, 95% CI: 54.8–93.0%), marking a more pronounced drop from its nursing-related performance compared to the other models.

### 3.4 Accuracy of image-based simulation test items

This study evaluated the performance of three multimodal large language models (Doubao, ERNIE 4.5 Turbo, and iFLYTEK Spark) on 80 image-based simulation questions (Table 3). DeepSeek-V3 was excluded from this analysis because it supports text-only input. Across all question types, iFLYTEK Spark achieved the highest accuracy, answering 49 of 80 questions correctly (61, 95% CI: 50.0–71.4%). Doubao and ERNIE 4.5 Turbo showed comparable overall performance, with accuracy rates of 48% (38/80, 95% CI: 37.2–58.9%) for both models. However, a chi-square test indicated that the differences in performance among the three models were not statistically significant (χ^2^ = 4.040, *p* = 0.133).

**Table 3:** The number of correct answers of Doubao, ERNIE 4.5 Turbo, and iFLYTEK Spark on the simulated image-based questions 2025.

Model	Question types	Right *N* (%)	Wrong *N* (%)	No response *N* (%)	χ^2^	*p*
Doubao	All questions	38 (48)	42 (52)	0 (0)	4.040	0.133
ERNIE 4.5 Turbo	All questions	38 (48)	40 (50)	2 (2)		
iFLYTEK Spark	All questions	49 (61)	24 (30)	7 (9)		
Doubao	A1	19 (43)	25 (57)	0 (0)	8.372	0.015
	A2	7 (33)	14 (67)	0 (0)		
	A3/A4	12 (80)	3 (20)	0 (0)		
	Case analysis questions	17 (53)	15 (47)	0 (0)	0.076	0.783
	Non-case analysis questions	27 (56)	21 (43)	0 (0)		
ERNIE 4.5 Turbo	A1	19 (43)	24 (55)	1 (2)	1.269	0.530
	A2	10 (48)	10 (48)	1 (4)		
	A3/A4	9 (60)	6 (40)	0 (0)		
	Case analysis questions	15 (47)	16 (50)	1 (3)	0.008	0.927
	Non-case analysis questions	23 (48)	24 (50)	1 (2)		
iFLYTEK Spark	A1	23 (52)	17 (39)	4 (9)	3.976	0.137
	A2	14 (67)	4 (19)	3 (14)		
	A3/A4	12 (80)	3 (20)	0 (0)		
	Case analysis questions	27 (84)	5 (16)	0 (0)	12.017	<0.01
	Non-case analysis questions	22 (46)	19 (40)	7 (14)		

A more detailed analysis of question subtypes revealed notable variations in model performance. Doubao performed differently across different question formats (χ^2^ = 8.372, *p* = 0.015). It achieved the highest accuracy on A3/A4 questions (80%, 12/15) and the lowest on A2 questions (33%, 7/21). The model performed slightly better on non-case analysis questions (56%, 27/48) than on case analysis questions (53%, 17/32), although this difference was not statistically significant (χ^2^ = 0.076, *p* = 0.783). ERNIE 4.5 Turbo followed a similar pattern, with its best performance also on A3/A4 questions (60%, 9/15). Performance was more consistent across other types, with accuracy rates of 43% on A1 and 48% on A2 questions. No significant difference was observed between its performance on case analysis (47%, 15/32) and non-case analysis questions (48%, 23/48; χ^2^ = 0.008, *p* = 0.927). iFLYTEK Spark showed the most distinct performance profile. Its high overall accuracy was largely driven by exceptional performance on case analysis questions, answering 84% (27/32) correctly. This was significantly higher than its performance on non-case analysis questions (46%, 22/48; χ^2^ = 12.017, *p* < 0.01). The model also achieved high accuracy on A3/A4 (80%, 12/15) and A2 questions (67%, 14/21), but exhibited a notably high “No response” rate for non-case analysis questions (14%, 7/48).

### 3.5 Comparison of accuracy between two test papers

A striking disparity was observed in the models’ performance on text-based questions versus image-based simulation test items (Table 4).

**Table 4:** Comparison of accuracy between the test items in the NNLE and the image-based simulation test items.

Model	Question types	Test items in the NNLE right *N* (%)	Image-based simulation test items right *N* (%)	χ^2^	*p*
DeepSeek-V3	All questions	223/240 (93)	N/A		
Doubao	All questions	227/240 (95)	38(48)	93.450	<0.01
ERNIE 4.5 Turbo	All questions	224/240 (93.3)	38(48)	84.935	<0.01
iFLYTEK Spark	All questions	221/240 (92)	49 (61)	43.267	<0.01
DeepSeek-V3	A1	101/112 (90)	N/A	182.966	<0.01
	A2	70/77 (91)	N/A	131.243	<0.01
	A3/A4	50/51 (98)	N/A	126.846	<0.01
	Case analysis questions	122/128 (95)	N/A	184.419	<0.01
	Non-case analysis questions	101/112 (90)	N/A	152.214	<0.01
	Nursing-related	209/225 (93)	N/A	236.093	<0.01
	Non-nursing	14/15 (93)	N/A	87.572	<0.01
Doubao	A1	103/112 (92)	19 (43)	44.105	<0.01
	A2	74/77 (96)	7 (33)	45.343	<0.01
	A3/A4	50/51 (98)	12 (80)	6.625	0.010
	Case analysis questions	123/128 (96)	17 (53)	43.214	<0.01
	Non-case analysis questions	103/112 (92)	27 (56)	28.132	<0.01
	Nursing-related	212/225 (94)	44 (55)	67.329	<0.01
	Non-nursing	15/15 (100)	/		
ERNIE 4.5 Turbo	A1	101/112 (90)	19 (43)	39.305	<0.01
	A2	72/77 (94)	10 (48)	25.433	<0.01
	A3/A4	51/51 (100)	9 (60)	22.440	<0.01
	Case analysis questions	123/128 (96)	15 (47)	52.292	<0.01
	Non-case analysis questions	101/112 (90)	23 (48)	3.573	0.059
	Nursing-related	212/225 (94)	38(48)	87.160	<0.01
	Non-nursing	12/15 (80)	/		
iFLYTEK Spark	A1	101/112 (90)	23 (52)	27.833	<0.01
	A2	72/77 (94)	14 (67)	11.062	<0.01
	A3/A4	51/51 (100)	12 (80)	10.686	0.001
	Case analysis questions	123/128 (96)	27 (84)	6.000	0.014
	Non-case analysis questions	101/112 (90)	22 (46)	38.168	<0.01
	Nursing-related	207/225 (92)	49 (61)	41.383	<0.01
	Non-nursing	14/15 (93)	/		

For text-based questions, all models demonstrated excellent and comparable performance, with overall accuracy rates ranging from 92 to 95%. DeepSeek-V3 achieved 93% (95% CI: 89.0–95.8%) accuracy on text items, while Doubao reached 95% (95% CI: 91.4–97.1%). In contrast, performance on the image-based simulation items dropped substantially for the multimodal models. DeepSeek-V3 was not evaluated on these items owing to its text-only input capability and is therefore marked as not applicable. Among the other three models, iFLYTEK Spark achieved the highest image-based accuracy at 61% (49/80), whereas Doubao and ERNIE 4.5 Turbo each recorded 48% (38/80). Chi-square tests revealed that the differences between text-based and image-based performance were statistically significant for all three models (all *p* < 0.01).

Further analysis of question subtypes revealed consistent patterns. On complex A3/A4 text-based questions, all models scored between 98 and 100%. Yet on the image-based versions of these same items, accuracies dropped substantially to 60–80%. Similarly, for case analysis questions, text-based performance exceeded 95% for most models, while image-based case analysis accuracy ranged from 47 to 84%.

## 4 Discussion

### 4.1 Overall model performance and clinical utility

Prior research has examined LLM performance on Chinese nursing examinations. Zong et al. (2024a, 2024b) found that ChatGPT failed to pass the NNLE over the 2017–2021 period, with accuracies ranging from 49 to 59%. The Chinese-developed language model Qwen-2.5 achieved an accuracy of 88.9% on the Chinese NNLE, while other Chinese models such as ERNIE Bot-3.5 attained 78.1%, iFLYTEK Spark 65% (Zhu et al., 2025). Xu L. H. et al. (2025) and Xu W. B. et al. (2025) further demonstrated that DeepSeek-V3 and Gemini 2.0 Pro exceeded 83% accuracy on the 2024 CNNLE. A longitudinal study by Zhan et al. (2026) tracking 15 LLMs from 2022 to 2025 showed a steep upward trajectory in performance, with top-tier models reaching 78.8% accuracy by 2025. Additionally, research has also revealed that iFLYTEK Spark achieved accuracies of 61 and 57.6% on the junior and intermediate levels of the China National Nursing Professional Qualification Examination, respectively (Li et al., 2025). Our primary finding is that all four models achieved text-based accuracies above 90%, substantially higher than previously reported for earlier LLM generations (e.g., ChatGPT-4.0: 69.1–71.9%) (Wu et al., 2024). It is generally consistent with those reported by Zong et al. for DeepSeek-R1 (91.59%) and by Wang et al. for DeepSeek-V3 (93%) on the National Medical Licensing Examination (Zong et al., 2026; Wang et al., 2025). From a nursing education perspective, this suggests that current mainstream Chinese LLMs already have the potential to assist nursing students with exam preparation and basic clinical knowledge retrieval (Mu et al., 2024). This result suggests that recent advancements in Chinese LLMs have substantially improved their performance on nursing licensure examinations. Notably, Doubao slightly outperformed the others with an accuracy of 94.6%, while DeepSeek-V3 and iFLYTEK Spark, despite certain limitations in some dimensions, still demonstrated stable overall performance.

However, this does not mean that these models can completely replace traditional review methods. Frame as an observation requiring future study: “Informal observations during testing suggested that some correct answers were accompanied by reasoning that contained logical gaps, though this was not systematically assessed. These observations suggest that caution is warranted when introducing LLMs into nursing education, and that their outputs should not be accepted uncritically (Mu et al., 2024; Li et al., 2026). Formal evaluation of reasoning quality and explanatory adequacy represents an important direction for future research. Resource constraints precluded inclusion of other influential models such as the Qwen series, GLM series, Hunyuan, and Kimi; future work should broaden the comparison to cover the full spectrum of Chinese LLMs relevant to nursing education.

### 4.2 Impact of model architecture differences on performance

These findings indicate that while current LLMs are highly proficient at answering text-only nursing questions, their ability to process multimodal information combining images with clinical text varies considerably and remains a significant challenge, particularly for DeepSeek-V3.

DeepSeek-V3 achieved high accuracy (92.9%) on pure text tests, falling within the range of 85.8 to 94.2% reported by Liu et al. (2026). Because the model accepts text input only, its performance on image-based items should be understood as not applicable rather than as incorrect. For the three multimodal models, accuracy on the simulated image tests dropped markedly to between 48 and 61%, well below their text-only performance of over 90%. This indicates that multimodal capabilities remain a significant challenge for LLMs, particularly in nursing contexts requiring clinical images interpretation (e.g., ECGs, wound photographs, and imaging data).

iFLYTEK Spark’s performance on image-based case analysis questions (84%) was significantly better than on non-case-analysis image questions (46%). Further analysis revealed that this discrepancy stemmed primarily from the model’s higher non-response rate when handling isolated images. That is, when an image was presented without a specific clinical context, the model was more likely to produce errors or fail to respond. This suggests that current multimodal models may rely more on textual semantic guidance than on independent visual understanding of clinical features.

### 4.3 Balance between specialized knowledge and general knowledge

Model performance differed between nursing-related and non-nursing-related questions. Doubao achieved 100% accuracy on non-nursing questions but 94% on nursing questions. This pattern may reflect differences in training data distribution rather than deficiencies in nursing knowledge. Deepseek-V3 maintained nearly identical accuracy across both categories, indicating a more balanced capability. In contrast, ERNIE 4.5 Turbo performed excellently on nursing-related questions (94%) but dropped to 80% on non-nursing questions, which was the largest decline among the four models. These results suggest that while all models are capable of handling exam-level content, their strengths differ across specialized and general domains. However, due to the small sample size of non-nursing questions (*n* = 15), these subgroup findings should be interpreted with caution and considered hypothesis-generating rather than confirmatory. Future studies with larger and more representative sets of non-nursing questions are needed to confirm these patterns.

These conclusions are based on only 15 non-nursing questions, a sample size insufficient for robust generalization. From a teaching perspective, this difference can inform a practical application strategy: when students need to look up interdisciplinary knowledge (e.g., pharmacological mechanisms or pathophysiological principles), Doubao may be the more reliable option; when the question focuses heavily on nursing operation standards or specialized nursing knowledge, ERNIE 4.5 Turbo is equally suitable. DeepSeek-V3 performed almost identically on both types of questions (93% vs. 93%), demonstrating a relatively balanced knowledge structure, which is an advantage for users who need to handle mixed-type questions.

### 4.4 Usability and alignment with practical application scenarios

From a user experience perspective, all four models were freely accessible and provided rapid responses (Tam et al., 2023; Xu L. H. et al., 2025; Xu W. B. et al., 2025; Zhu et al., 2026), which lowered the barrier for nursing educators and students. Measured response times ranged from 8.7 to 12.4 s per question. DeepSeek-V3’s higher session capacity (120 questions) may facilitate exam simulation and large-scale practice. In contrast, Doubao imposed a limit on the number of questions per session, which may be inconvenient in actual teaching.

However, ease of use was not only a technical metric; it also involved the reliability of the answers, the comprehensibility of the explanations, and the mastery of nursing-specific terminology. During testing, we observed differences in answer styles across models: some models tended to give brief, direct answers, while others provided detailed reasoning processes. Although these qualitative observations were not systematically quantified in the present study, for nursing students, the latter might hold greater educational value, as it helps them understand “why this answer is chosen” rather than simply “which answer to choose”.

### 4.5 Advantages and limitations

Strengths of this study include the systematic comparison of four leading Chinese LLMs on the NNLE, the inclusion of image-based questions, and detailed subgroup analyses across question types, case status, and disciplinary content.

However, several limitations of this study should be acknowledged. First, only 80 image-based simulation questions were used, which limits the reliability and external validity of the findings. Second, we used standard answers to determine correctness. In the nursing field, however, some questions are inherently controversial, and the optimal answer may vary with clinical context; thus, a binary correct/incorrect judgment may oversimplify the issue. Third, model performance can change significantly with version updates, so our conclusions are limited to the model versions tested in January 2026. This temporal constraint limits the generalisability of our results to ongoing educational use. Fourth, this study adopted a single-question strategy without repeated sampling of the model’s output, which precludes any assessment of the model’s stability. As prior work has shown that LLMs can vary considerably when answering the same question across sessions, with consistency dropping below 87% even on high-accuracy items (Xu L. H. et al., 2025; Xu W. B. et al., 2025). Without repeated testing, we cannot determine whether a correct answer reflects genuine understanding or stochastic output. Fifth, the small sample size of non-nursing questions (*n* = 15) limits the generalizability of our findings regarding domain-specific performance differences. Future studies should consider: (a) administering each question multiple times (≥3) to evaluate intra-model consistency and report variability alongside accuracy; (b) tracking performance longitudinally across version updates to quantify drift; and (c) using standardised prompts with temperature set to 0 to reduce random variation, as recommended by Xu L. H. et al. (2025) and Xu W. B. et al. (2025).

The image-based questions used in this study were derived from simulation materials rather than official NNLE examination items. Although we made efforts to ensure the content, format, and difficulty of these simulated questions were comparable to those of the official examination, we cannot guarantee that they perfectly replicate the characteristics of authentic image-based NNLE questions. The inability to use official image-based items is a constraint imposed by copyright restrictions on the release of examination materials. Future research should collaborate with examination authorities to obtain access to authentic image-based items or develop larger, validated multimodal benchmarking datasets that can be shared across studies to facilitate comparability.

All materials used in this study were employed in compliance with copyright exemptions for academic research. we acknowledge the potential for data leakage, as the examination materials were published in 2025 and the commercial models were evaluated in January 2026. It is possible that portions of the test questions had entered the training corpora or retrieval systems of these models. The readers should interpret the high text-based accuracy results with this limitation in mind. To rigorously assess true inferential capacity, future studies should employ prospectively designed, non-disclosed question banks that are guaranteed to be absent from any training data.

### 4.6 Implications for nursing education practice

Based on the above findings, we offer several recommendations for nursing educators who may be exploring the integration of LLMs into their teaching. First, these models could serve as useful tools to help teachers prepare classroom teaching materials and help students strengthen their understanding of medical knowledge, but they should not be regarded as a primary or authoritative source of knowledge (Zong et al., 2024a, 2024b). Educators might encourage students to cross-check model outputs against standard textbooks and clinical protocols, thereby promoting critical appraisal skills. Second, in teaching scenarios involving image recognition (e.g., ECG interpretation, wound assessment, or analysis of imaging results), current model performance is insufficient to replace instructor guidance, especially for high-risk clinical decisions. Third, given that the four models show different relative strengths, educators and students could choose among them according to the specific task, while remaining aware of the limitations observed in this study. For example, DeepSeek-V3 may be suitable for text-only practice; iFLYTEK Spark performed better on image-based case analysis; Doubao may showed an advantage in general knowledge. However, these observations are preliminary and should be verified in authentic educational settings before any practical recommendations are drawn.

## 5 Conclusion

In summary, DeepSeek-V3, Doubao, ERNIE 4.5 Turbo, and iFlytek Spark all models achieved text-based accuracy above 90%, suggesting potential as educational support tools. However, the performance of all models declined markedly on image-based questions, indicating that multimodal capability remains a major bottleneck. The models showed different strengths in nursing-specific versus general knowledge, and users can select an appropriate tool based on their actual needs. This study provides preliminary empirical evidence for nursing educators considering the integration of LLMs into teaching practices. Nevertheless, caution is warranted when applying these models, and their current technological limitations should be recognized.

## Data Availability

The original contributions presented in the study are included in the article/supplementary material, further inquiries can be directed to the corresponding author.
